# Circuit network theory of *n*-horizontal bridge structure

**DOI:** 10.1038/s41598-022-09841-2

**Published:** 2022-04-13

**Authors:** Xin-Yu Fang, Zhi-Zhong Tan

**Affiliations:** Department of Physics, Nantong University, Nantong, 226019 China

**Keywords:** Complex networks, Statistical physics, Applied mathematics

## Abstract

This research investigates a complex *n* order cascading circuit network with embedded horizontal bridge circuits with the N-RT method. The contents of the study include equivalent resistance analytical formula and complex impedance characteristics of the circuit network. The research idea is as follows. Firstly the equivalent model of *n*-order resistance network is established, and a fractional difference equation model is derived using Kirchhoff’s law. Secondly, the equivalent transformation method is employed to transform the fractional equation into a simple linear difference equation, and its particular solution is computed. Then the solution to the difference equation is used to derive the effective resistance of the resistance network of the embedded horizontal bridge circuit, and various special cases of equivalent resistance formula are analyzed and the correctness of the analysis model gets verified. Finally, as an expanded application, the equivalent complex impedance of *LC* network is studied, and Matlab drawing tool is employed to offer the equivalent impedance with various variables of the graph. Our results provide new research ideas and theoretical basis for relevant scientific researches and practical applications.

## Introduction

Since Kirchhoff founded the circuit foundation theory in 1845^1^, people has been exposed to a new era of circuit exploration. The resistance network research and its applications have been greatly developed^2–28^. Due to the intuitive advantages of resistance network model, the research of resistor network has good practical values. For hundreds of years, researchers have been exploring the mystery of the circuit. Venezian explored the resistance between two points on the grid^2^, and then new research ideas were proposed; Atkinson and Steenwijk studied the infinite resistive circuit^3^; The Green’s function technique^4^ for studying the Infinity Network founded by Cserti opened the door to the study of infinite circuit networks^5–8^. The Laplace matrix method created by Professor Wu in 2004^9^ let the research of circuit networks reach a new level, including finite and infinite resistance networks. Subsequently, more and more scholars had been devoted to the exploration of the world of resistive networks^10–13^. In 2011, Professor Tan advanced the more widely-used Recursion–Transform method (RT)^14–17^, which is applied to solve a variety of complex *m* × *n* order networks and equivalent resistance problems with different complex boundary conditions^18–25^. Tan’s method can be employed to study arbitrary circuit network, and therefore the complex boundary network problems are well addressed. Naturally, the method provides the greatest possibility for circuit network research as well.

**Figure 1:**
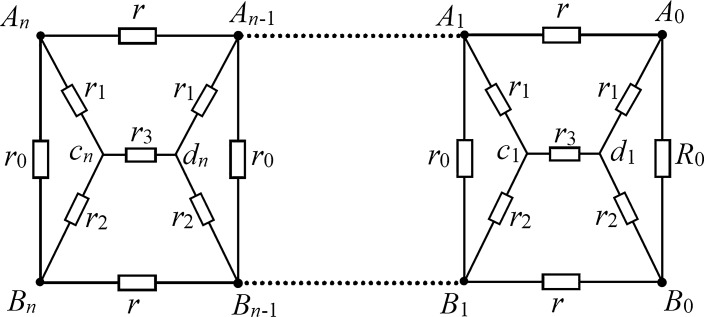
An *n*-bridge circuit network model with 6 different resistance elements.

In recent years, Tan’s RT methods and techniques have been used to solve many different types of resistor network models. But as realistic problems may be more complex, many network models containing complex internal structures have not been resolved. In recent studies, some new progress has been made in the study of complex N-order circuit networks. For example, literatures^27–32^ calculated the electrical properties of N-order networks of multiple complex structures respectively, and literatures^27–29,31,32^ studied complex impedance properties of *LC* circuit networks with different structures respectively. The study of these *LC* circuit network models is conducive to the research of the popular problem of Metagratings^33,34^. In addition, the authors in Ref.^27^ named a general recurrence transformation method for studying the N-order resistor network model proposed in Ref.^14^ as the N-RT method. The N-RT method is also used in the present study in details for the resistance network of the embedded horizontal bridge circuit structure, aiming at study its equivalent resistance analysis formula and the complex impedance characteristics of LC network, as shown in Fig. 1. It contains 6 different resistance elements, which is a complex and versatile network model that is never discussed.

Considering the general *n*-order horizontal bridge circuit network model (as shown in Fig. 1), the upper and lower boundary resistance is *r* and the resistance on the vertical edge is *r*_0_, the resistance elements in the bridge circuit are *r*_1_, *r*_2_ and *r*_3_, and the load resistance *R*_0_ is limited to the right boundary. Obviously, the resistance network in Fig. 1 is composed of six different resistance elements, which means that it is a multi-value network model. This paper focuses on the analytical expression of the effective resistance and the analytical expression of the complex impedance between *A*_*n*_ and *B*_*n*_ in the network in Fig. 1, obtains the theoretical results of the concise structure, and reveals the variation characteristics of the complex impedance under different conditions combined with the Matlab drawing tool.

## Results

Considering the complex network of Fig. 1, the positional arrangement of the node *A*_*k*_ and *B*_*k*_ in the network model are shown in Fig. 1. The N-RT technique^14,27^ is used to study the resistive network and a simple analytical formula of resistance formula is found as follows,

1 $$R_{n} (A_{n} B_{n} ) = \lambda - \alpha \beta \frac{{F_{n} + (R_{0} - \lambda )F_{n - 1} }}{{F_{n + 1} + (R_{0} - \lambda )F_{n} }},$$

where *R*_0_ is the any resistance of the right edge, as well as

2 $$\lambda = \frac{c + 1}{{d + 1}}r_{0} ,\;\alpha \beta = \frac{{(r_{1} + r_{2} )(d - c) - (c + 1)r_{0} }}{{(d + 1)^{2} }}r_{0} ,$$

3 $$F_{n} = \frac{{\alpha^{n} - \beta^{n} }}{\alpha - \beta },$$

and, along with that is

4 $$\begin{gathered} \alpha = \frac{{(r_{1} + r_{2} )d + cr_{0} + \sqrt {[(r_{1} + r_{2} )d + cr_{0} ]^{2} - 4(r_{1} + r_{2} )(d - c)r_{0} + 4(c + 1)r_{0}^{2} } }}{2(d + 1)}, \hfill \\ \beta = \frac{{(r_{1} + r_{2} )d + cr_{0} { - }\sqrt {[(r_{1} + r_{2} )d + cr_{0} ]^{2} - 4(r_{1} + r_{2} )(d - c)r_{0} + 4(c + 1)r_{0}^{2} } }}{2(d + 1)}. \hfill \\ \end{gathered}$$

with

5 $$c = \frac{(a + b)r}{{ar_{2} + br_{1} }},\;d = \frac{{(b + a)rr_{1} r_{2} + r_{0} ab}}{{r_{1} r_{2} (ar_{2} + br_{1} )}} - \frac{{r_{0} r_{3} }}{{r_{1} r_{2} }},$$

6 $$a = (2r_{1} + r_{3} + r)r_{2} + r_{1} r_{3} ,\;b = (2r_{2} + r_{3} + r)r_{1} + r_{2} r_{3} .$$

## Methods

### Modeling recursive equation

**Figure 2:**
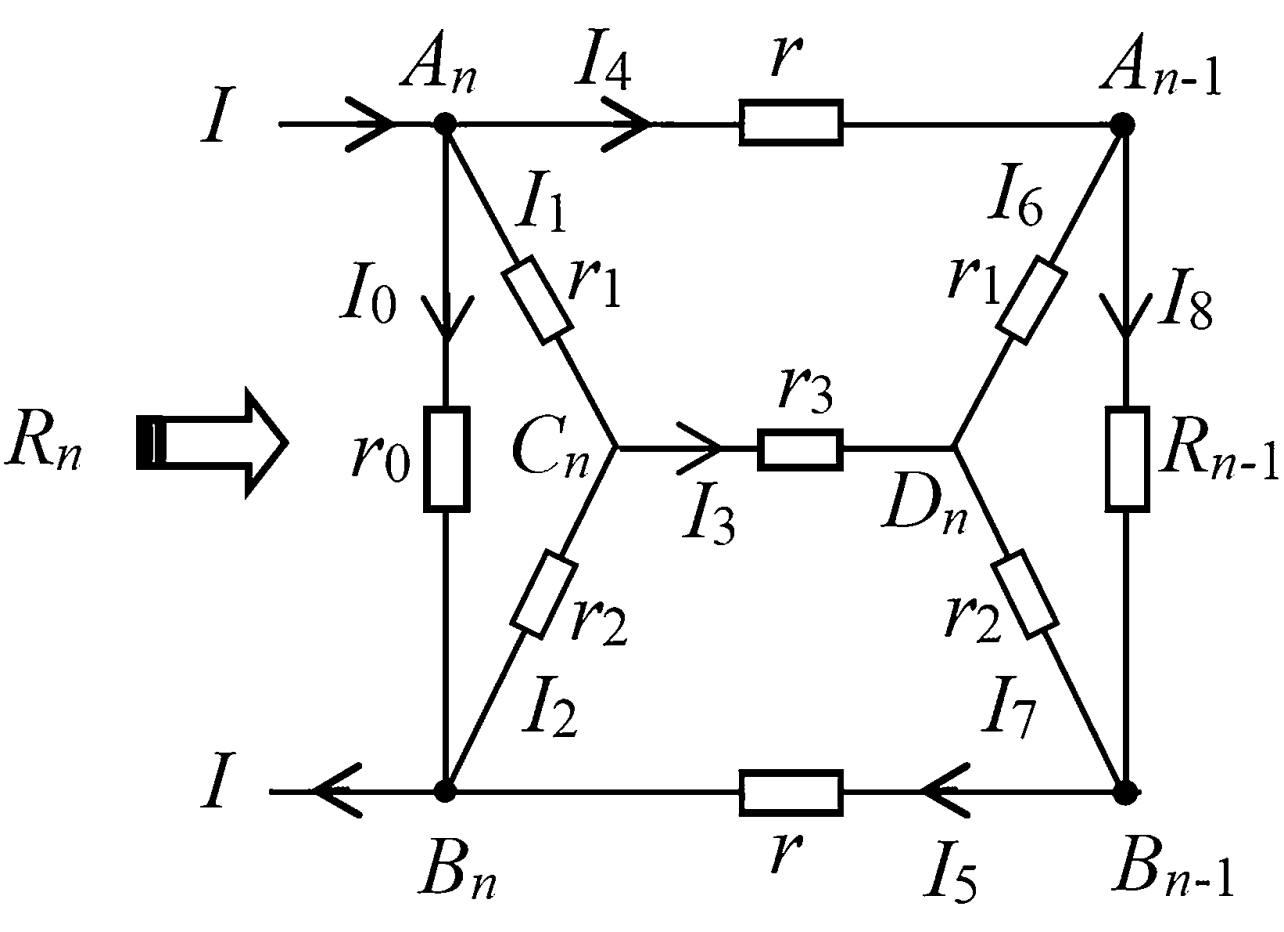
Equivalent model of a bridge circuit network with current parameters.

We use the N-RT technique to study the circuit network according to Fig. 1, assuming the effective resistance between the left edge *A*_*n*_ and the *B*_*n*_ is *R*_*n*_, and the equivalent resistance between the two nodes at the left edge of the *n* − 1 network is R_n−1_. Therefore, the network in Fig. 1 can be equivalently simplified to the model in Fig. 2. It is the key to solving the problem how to build the recursive relations between *A*_*n*_ and *B*_*n*_. Here, we use the circuit theory to establish the relationship between *R*_*n*_ and *R*_*n*-1_. The equivalent model of bridge circuit network with current parameters is shown in Fig. 2.

In Fig. 2, we can obtain a series of current equations using the circuit voltage theory,

7 $$I_{1} r_{1} + I_{2} r_{2} - I_{0} r_{0} = 0,$$

8 $$I_{4} r + I_{6} r_{1} - I_{3} r_{3} - I_{1} r_{1} = 0,$$

9 $$I_{3} r_{3} + I_{7} r_{2} + I_{5} r - I_{2} r_{2} = 0,$$

10 $$I_{6} r_{1} + I_{7} r_{2} - I_{8} R_{n - 1} = 0.$$

In Fig. 2, we also obtain the node current equation through the node current law,

11 $$I_{1} + I_{4} + I_{0} = I,$$

12 $$I_{2} + I_{5} + I_{0} = I,$$

13 $$I_{3} = I_{1} - I_{2} = I_{7} - I_{6} ,$$

14 $$I_{8} = I_{4} - I_{6} = I_{5} - I_{7} .$$

Through the multiple substitution simplification of the above current equations, we can eliminate the parameters of $I_{1} - I_{8}$ in Eqs. (7)–(14), and finally obtain the relationship between $I_{0} \propto I$ and $R_{n - 1}$(solving the above eight equations is complex, and the derivation process is omitted here), then use Ohm’s law $R_{n} = U_{{A_{n} B_{n} }} /I = I_{0} r_{0} /I$, the resistance relation equation is as follows

15 $$R_{n} = \frac{{c(r_{1} + r_{2} )r_{0} + r_{0} (c + 1)R_{n - 1} }}{{(r_{1} + r_{2} )d - r_{0} + (d + 1)R_{n - 1} }}.$$

where *c, d* are constants determined by the resistance in Eqs. (7)–(10), which are given by Eqs. (5) and (6). Thus, the analytical formula of equivalent resistance can be studied based on this recurrence equation.

### Solving the equation by substitution of variables

How to solve Eq. (15) is the key to solving the problem. Fortunately, Ref.^14^ established a good method for solving the complex difference equations, and we solve the Eq. (15) by variable substitution method, assuming that there is a sequence $\{ x_{n} \}$ to be determined, and we make the following transformation:

16 $$R_{n} = \frac{{x_{n + 1} }}{{x_{n} }} - \frac{{(r_{1} + r_{2} )d - r_{0} }}{d + 1}.$$

We suggest that the initial term be $x_{0} = 1$, from Eq. (16), we can determine $x_{1}$, so we have

17 $$x_{0} = 1,\;x_{1} = R_{0} + \frac{{(r_{1} + r_{2} )d - r_{0} }}{d + 1}.$$

Replace Eq. (16) and its recurrence formula $R_{n - 1}$ into Eq. (15) and we can obtain it through algebraic calculation

18 $$x_{n + 1} = \frac{{(r_{1} + r_{2} )d + cr_{0} }}{d + 1}x_{n} - \frac{{(r_{1} + r_{2} )(d - c)r_{0} - (c + 1)r_{0}^{2} }}{{(d + 1)^{2} }}x_{n - 1} .$$

In order to solve Eq. (18), we first need to solve its characteristic root, by Ref.^14^ we know its characteristic equation is obtained from Eq. (18)

19 $$x^{2} = \frac{{(r_{1} + r_{2} )d + cr_{0} }}{d + 1}x - \frac{{(r_{1} + r_{2} )(d - c)r_{0} - (c + 1)r_{0}^{2} }}{{(d + 1)^{2} }}.$$

So, let $\alpha$ and $\beta$ be the two roots of the characteristic Eq. (19), and (18) can be transformed into

20 $$x_{n + 1} = \left( {\alpha + \beta } \right)x_{n} - \alpha \beta x_{n - 1} .$$

Using the method provided in Ref.^14^ to solve the difference Eq. (20), we get

21 $$x_{n} = \frac{1}{\alpha - \beta }\left[ {\left( {x_{1} - \beta x_{0} } \right)\alpha^{n} - \left( {x_{1} - \alpha x_{0} } \right)\beta^{n} } \right].$$

The initial value Eq. (17) is substituted into Eq. (21)

22 $$x_{n} = \frac{1}{\alpha - \beta }\left[ {\left( {R_{0} + \frac{{(r_{1} + r_{2} )d - r_{0} }}{d + 1} - \beta } \right)\alpha^{n} - \left( {R_{0} + \frac{{(r_{1} + r_{2} )d - r_{0} }}{d + 1} - \alpha } \right)\beta^{n} } \right].$$

Since $\alpha ,\beta$ are the two roots of Eq. (19), the following simple relationship can be obtained from (19)

23 $$\alpha + \beta = \frac{{(r_{1} + r_{2} )d + cr_{0} }}{d + 1} = \frac{{(r_{1} + r_{2} )d - r_{0} }}{d + 1} + \lambda ,$$

where $\lambda = (c + 1)r_{0} /(d + 1)$ is defined in Eq. (2). By substituting Eq. (23) into Eq. (22), we obtain

24 $$x_{n} = \frac{1}{\alpha - \beta }\left[ {\left( {R_{0} + \alpha - \lambda } \right)\alpha^{n} - \left( {R_{0} + \beta - \lambda } \right)\beta^{n} } \right].$$

Furthermore, the expression Eq. (24) can be simplified by the function of $F_{n}$

25 $$x_{n} = F_{n + 1} + (R_{0} - \lambda )F_{n} ,$$

of which $F_{n} = {{(\alpha^{n} - \beta^{n} )} \mathord{\left/ {\vphantom {{(\alpha^{n} - \beta^{n} )} {(\alpha - \beta )}}} \right. \kern-\nulldelimiterspace} {(\alpha - \beta )}}$. According to Eq. (23) $[(r_{1} + r_{2} )d - r_{0} ]/(d + 1) = \alpha + \beta - \lambda$, which is a deformation of the constant term in Eq. (16). So, using Eqs. (20) and (23), Eq. (16) can be written as

26 $$R_{n} = \lambda { + }\frac{{x_{n + 1} }}{{x_{n} }} - (\alpha + \beta ) = \lambda - \alpha \beta \frac{{x_{n - 1} }}{{x_{n} }}.$$

The purpose of transforming Eq. (16) into Eq. (26) is to make the expression more concise and beautiful. Substituting Eq. (25) and its recurrence formula $x_{n - 1}$ into Eq. (26), yielding

27 $$R_{n} = \lambda - \alpha \beta \frac{{F_{n} + (R_{0} - \lambda )F_{n - 1} }}{{F_{n + 1} + (R_{0} - \lambda )F_{n} }}.$$

Since $\alpha ,\beta$ are the two roots of Eq. (19), it is easy to obtain from Eq. (19)

28 $$\alpha \beta = \frac{{(r_{1} + r_{2} )(d - c)r_{0} - (c + 1)r_{0}^{2} }}{{(d + 1)^{2} }}.$$

Therefore, Eq. (1) is verified to be correct. The above calculation process is rigorous and self-consistent.

## Discussion

### Analysis of special cases

Figure 1 is a general circuit network model, and formula (1) is a general equivalent resistance result. Since the network in Fig. 1 contains six independent parameters, it is a multi-functional and multi-purpose network, including a variety of network model topologies, which has important application values. As several interesting applications, a series of special results of formula (1) are given below.

*Special case-1 *$r_{3} = r_{1}$*.* Considering the network of Fig. 1, when $r_{3} = r_{1}$, through Eqs. (5) and (6), we get

29 $$\begin{gathered}  a = 3r_{1} r_{2} + r_{1}^{2} + rr_{2} ,\;b = 3r_{1} r_{2} + r_{1}^{2} + rr_{1} , \hfill \\ c = \frac{(a + b)r}{{ar_{2} + br_{1} }},\;d = \frac{{(b + a)rr_{1} r_{2} + r_{0} ab}}{{r_{1} r_{2} (ar_{2} + br_{1} )}} - \frac{{r_{0} }}{{r_{2} }}, \hfill \\ \end{gathered}$$

30 $$\lambda = \frac{c + 1}{{d + 1}}r_{0} ,\;\alpha \beta = \frac{{r_{0}^{2} }}{{(d + 1)^{2} }}.$$

So, using Eq. (1), we have

31 $$R_{n} (A_{n} B_{n} ) = \lambda - \left( {\frac{{r_{0} }}{d + 1}} \right)^{2} \frac{{F_{n} + (R_{0} - \lambda )F_{n - 1} }}{{F_{n + 1} + (R_{0} - \lambda )F_{n} }},$$

where $\lambda = (c + 1)r_{0} /(d + 1)$ is given by (30), and $\alpha$, $\beta$ are still given by Eq. (4), but their parameters *a, b, c* and *d* are given by Eq. (29).

**Figure 3:**
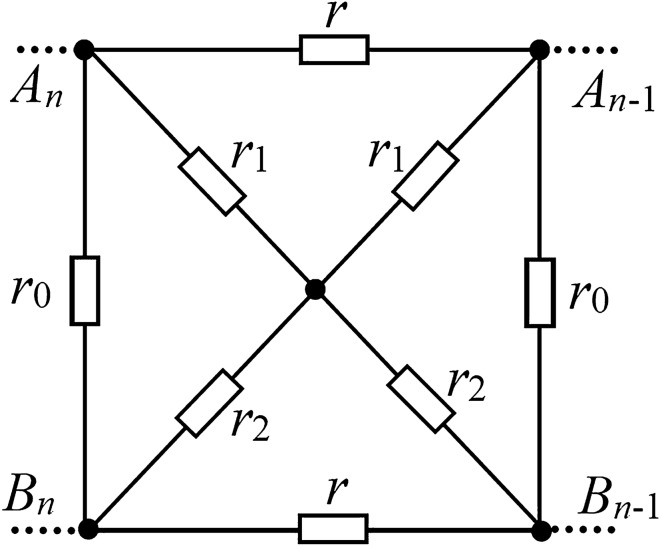
Subnet diagram with embedded cross resistor.

*Special case-2 *$r_{3} = 0$*.* Considering the network of Fig. 1, when $r_{3} = 0$, Fig. 1 changes to the structure of Fig. 3, which is obtained from Eq. (5)

32 $$\begin{gathered} a = (2r_{1} + r)r_{2} ,\;b = (r + 2r_{2} )r_{1} , \hfill \\ c = \frac{{r(r_{2} + r_{1} ) + 4r_{2} r_{1} }}{{r(r_{2}^{2} + r_{1}^{2} ) + 2r_{1} r_{2} (r_{2} + r_{1} )}}r,\;d = \frac{{(b + a)rr_{1} r_{2} + r_{0} ab}}{{r_{1} r_{2} (ar_{2} + br_{1} )}}. \hfill \\ \end{gathered}$$

So, using Eq. (1), we have

33 $$R_{n} (A_{n} B_{n} ) = \lambda - \left( {\frac{{r_{0} }}{d + 1}} \right)^{2} \frac{{F_{n} + (R_{0} - \lambda )F_{n - 1} }}{{F_{n + 1} + (R_{0} - \lambda )F_{n} }},$$

where $\lambda = (c + 1)r_{0} /(d + 1)$ is given by (30), and $\alpha$, $\beta$ are still given by Eq. (4), but their parameters *a, b, c* and *d* are given by Eq. (32).

*Special case-3 *$r_{1} = r_{2} = r_{3} = r$*.* For the network of Fig. 1, when $r_{1} = r_{2} = r_{3} = r$, we get from Eq. (6)

34 $$b = a = 5r^{2} .$$

Substituting $a = b$ into Eq. (5), we obtain

35 $$d = 1 + \frac{{3r_{0} }}{2r},\;c = 1,$$

and

36 $$\lambda = \frac{2}{d + 1}r_{0} = \frac{{4rr_{0} }}{{3r_{0} + 4r}},\;\alpha \beta = \left( {\frac{2h}{{4h + 3}}} \right)^{2} .$$

Substituting (34) and (35) into (4), we obtain

37 $$\begin{gathered} \alpha = 2r\frac{{r + 2r_{0} + \sqrt {(r + 3r_{0} )(r + r_{0} )} }}{{4r + 3r_{0} }}, \hfill \\ \beta = 2r\frac{{r + 2r_{0} - \sqrt {(r + 3r_{0} )(r + r_{0} )} }}{{4r + 3r_{0} }}. \hfill \\ \end{gathered}$$

Thus, substituting (36) into (1), we have

38 $$R_{n} (A_{n} B_{n} ) = \lambda - \left( {\frac{{2rr_{0} }}{{3r_{0} + 4r}}} \right)^{2} \frac{{F_{n} + (R_{0} - \lambda )F_{n - 1} }}{{F_{n + 1} + (R_{0} - \lambda )F_{n} }},$$

where $F_{n} = {{(\alpha^{n} - \beta^{n} )} \mathord{\left/ {\vphantom {{(\alpha^{n} - \beta^{n} )} {(\alpha - \beta )}}} \right. \kern-\nulldelimiterspace} {(\alpha - \beta )}}$, and $\lambda$ is given by (36).

*Special case-4 *$r_{1} = r_{2} = r_{0}$ and $r_{3} = r$. For the network of Fig. 1, when $r_{1} = r_{2} = r_{0}$ and $r_{3} = r$, from Eqs. (5) and (6) we get

39 $$b = a = (2r_{0} + 3r)r_{0} ,\;c = \frac{r}{{r_{0} }} = h,\;d = \frac{1}{2}(2 + 3h),$$

40 $$\lambda = 2r_{0} \frac{h + 1}{{4 + 3h}},\;\alpha \beta = \frac{{r_{0}^{2} }}{{(d + 1)^{2} }} = \left( {\frac{{2r_{0} }}{4 + 3h}} \right)^{2} .$$

where $h = r/r_{{0}}$. Substituting Eq. (39) into (4), we get

41 $$\alpha = 2r_{0} \frac{{1 + 2h + 2\sqrt {h(h + 1)} }}{4 + 3h},\;\beta = 2r_{0} \frac{{1 + 2h + 2\sqrt {h(h + 1)} }}{4 + 3h}.$$

So, if it is substituted in formula (1), we can get

42 $$R_{n} (A_{n} B_{n} ) = \lambda - \left( {\frac{{2r_{0} }}{4 + 3h}} \right)^{2} \frac{{F_{n} + (R_{0} - \lambda )F_{n - 1} }}{{F_{n + 1} + (R_{0} - \lambda )F_{n} }},$$

of which $\lambda$ is given by Eq. (40).

**Figure 4:**
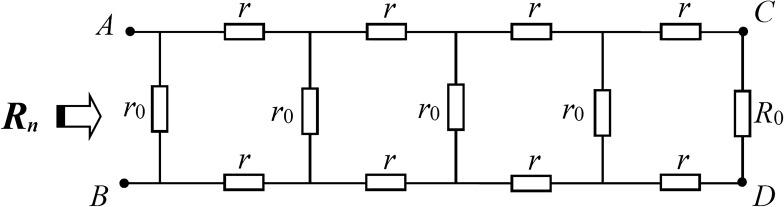
*n*-order resistor network with arbitrary right boundary.

*Special case-5 *$r_{1} = r_{2} = r_{3} \to \infty$*.* When $r_{1} = r_{2} = r_{3} \to \infty$, the resistor network in Fig. 1 becomes a simple network model in Fig. 4, which is a common problem in circuit theory. Take the limit and substitute it into formula (4) and we can obtain

43 $$\begin{gathered} \alpha = r + r_{0} + \sqrt {2rr_{0} + r^{2} } , \hfill \\ \beta = r + r_{0} - \sqrt {2rr_{0} + r^{2} .} \hfill \\ \end{gathered}$$

So, if they are substituted in formula (1), we can get

44 $$R_{n} (A_{n} B_{n} ) = r_{0} \left( {1 - r_{0} \frac{{F_{n} + (R_{0} - r_{0} )F_{n - 1} }}{{F_{n + 1} + (R_{0} - r_{0} )F_{n} }}} \right).$$

The result (44) is completely consistent with the special results in Refs.^14,25–27^, which indirectly verifies the correctness of the conclusion in this paper.

*Special case-6 *$n \to \infty$*.* When $n \to \infty$, from Eq. (4), we know $0 < \beta /\alpha < 1$, so $\mathop {\lim }\limits_{n \to \infty } \left( {\frac{\alpha }{\beta }} \right)^{n} = 0$, and hence we get from Eq. (1)

45 $$R_{{A_{n} B_{n} }} \left( {n \to \infty } \right) = \lambda - \beta .$$

Substituting Eq. (4) into Eq. (45), we have

46 $$R_{{A_{n} B_{n} }} \left( {n \to \infty } \right) = \frac{{(c + 2)r_{0} - (r_{1} + r_{2} )d + \sqrt \Delta }}{2(d + 1)},$$

where $$\Delta = [(r_{1} + r_{2} )d + cr_{0} ]^{2} - 4(r_{1} + r_{2} )(d - c)r_{0} + 4(c + 1)r_{0}^{2}$$.

*Special case-7 *$R_{0} = \lambda - \beta$*.* When $R_{0} = \lambda - \beta$, if we substitute it into Eq. (1) and simplify it, we get

47 $$R_{{A_{n} B_{n} }} \left( {n \in N} \right) = \lambda - \beta = \frac{{(c + 2)r_{0} - (r_{1} + r_{2} )d + \sqrt \Delta }}{2(d + 1)}.$$

Although the expressions of Eqs. (46) and (47) are the same, their meanings are different. For example, there is $n \to \infty$ in Eq. (46), but only $R_{0} = \lambda - \beta$ existing in Eq. (47).

### Research on *n-LC* impedance network of bridge circuit

**Figure 5:**
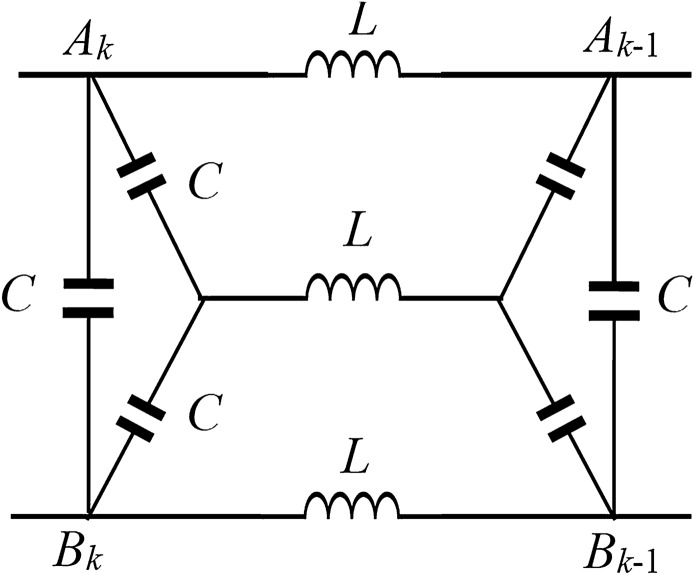
LC circuit sub network model with bridge circuit structure.

The research results of the resistor network in this paper can also be applied to the *LC* complex impedance network shown in Fig. 5 through the following variable substitution techniques. Assume that the element frequency of AC is ω, and we can carry out the transformation of resistance and complex impedance as follows

48 $$\begin{gathered} R_{0} = qr_{1} = q/j\omega C,\;r = r_{3} = j\omega L, \hfill \\ r_{1} = r_{0} = r_{2} = (j\omega C)^{ - 1} ,\;h = r/r_{1} = - \omega^{2} LC = - x. \hfill \\ \end{gathered}$$

Substituting (48) into Eqs. (2), (4) and (5), we can obtain

49 $$h = - x, d = 1 - \frac{3x}{2},$$

50 $$\delta = \frac{\alpha }{{r_{0} }} = 2\frac{{1 - 2x + 2\sqrt {x(x - 1)} }}{4 - 3x},\;\eta = \frac{\beta }{{r_{0} }} = 2\frac{{1 - 2x - 2\sqrt {x(x - 1)} }}{4 - 3x},$$

51 $$\lambda = r_{0} \cdot \frac{{2\left( {1 - x} \right)}}{4 - 3x},\;\alpha \beta = \frac{{4r_{0}^{2} }}{{(4 - 3x)^{2} }}.$$

The equivalent complex impedance formula can be obtained by substituting Eqs. (50) and (51) into formula (1)

52 $$\frac{{Z_{n} (A_{n} B_{n} )}}{{r_{0} }} = \frac{2(1 - x)}{{4 - 3x}} - \frac{4}{{(4 - 3x)^{2} }} \cdot \frac{{\delta^{n} - \eta^{n} + \left[ {q - \frac{2(1 - x)}{{4 - 3x}}} \right]\left( {\delta^{n - 1} - \eta^{n - 1} } \right)}}{{\delta^{n + 1} - \eta^{n + 1} + \left[ {q - \frac{2(1 - x)}{{4 - 3x}}} \right]\left( {\delta^{n} - \eta^{n} } \right)}},$$

where $\delta$ and $\eta$ are defined in Eq. (50), and *q* is a constant determined by the load.

According to the eigenvalue expression (50), we need to discuss and analyze many cases in which the eigenvalues are real numbers and imaginary numbers respectively.

**Figure 6:**
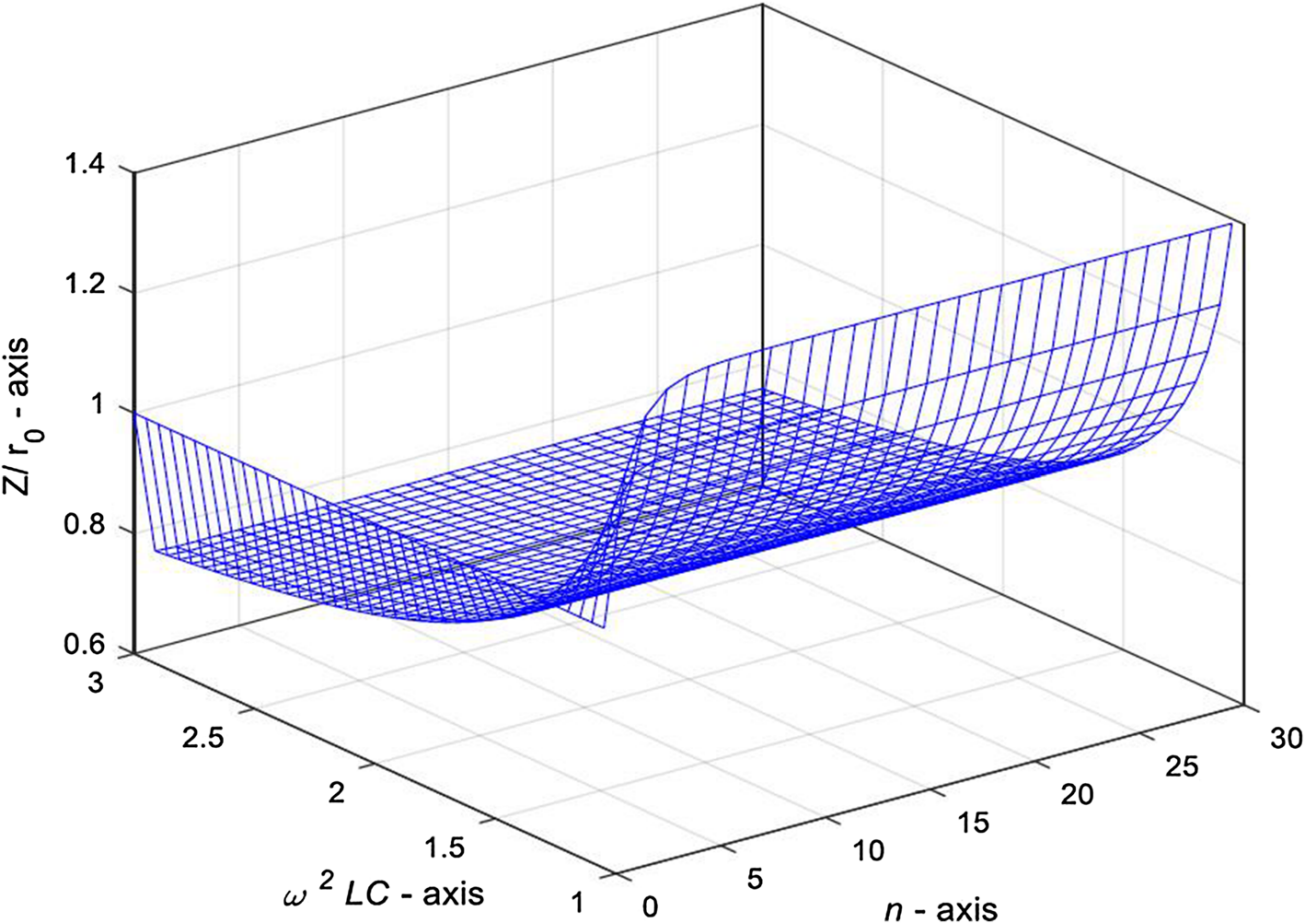
3D graph of complex impedance ${Z \mathord{\left/ {\vphantom {Z r}} \right. \kern-\nulldelimiterspace} r}_{0}$ varying with variables $\omega^{2} LC$ and $\mathrm{n}$, in case of $1 \le x \le 2$ and $q = 1$.

*The case of* $\omega^{2} LC \ge 1$**.** When $\omega^{2} LC = x \ge 1$, and its characteristic roots $\delta$ and $\eta$ are real numbers, the complex impedance expression is the structure of Eq. (52). Next, we draw a 3D characteristic curve with MATLAB to reveal the variation law of complex impedance when the characteristic root is real numbers, the change curve is shown in Fig. 6.

Figure 6 shows that when $1 \le x \le 2$ and $q = 1$, the complex impedance ${Z \mathord{\left/ {\vphantom {Z r}} \right. \kern-\nulldelimiterspace} r}_{0}$ in Eq. (52) changes irregularly with the increase of $\omega^{2} LC$ and $n$. It should be noted that there is no oscillation in this case. Next, let’s explore the transformation law of the complex impedance when *n* is the given value and *q* is the variable.

**Figure 7:**
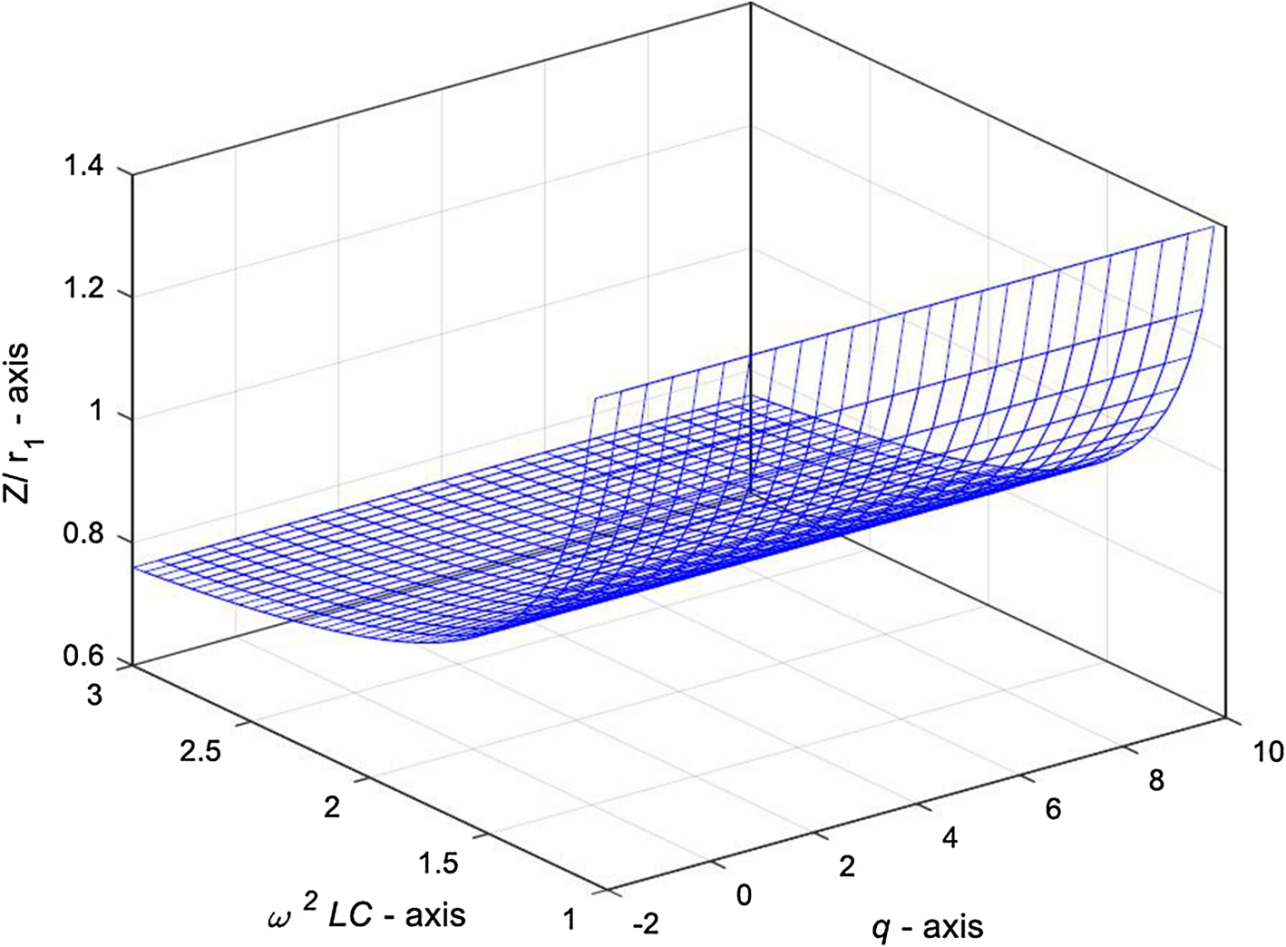
3D graph of complex impedance ${Z \mathord{\left/ {\vphantom {Z r}} \right. \kern-\nulldelimiterspace} r}_{0}$ varying with variables $\omega^{2} LC$ and $q$, in case of $1 \le x \le 2$ and $n = 30$.

Figure 7 shows that when $1 \le x \le 2$ and $n = 30$, the complex impedance ${Z \mathord{\left/ {\vphantom {Z r}} \right. \kern-\nulldelimiterspace} r}_{0}$ increases with the increase of $\omega^{2} LC$ based on the Eq. (52). In the interval $\omega^{2} LC \ge 1$, when $\omega^{2} LC \to 1$, the complex impedance ${Z \mathord{\left/ {\vphantom {Z r}} \right. \kern-\nulldelimiterspace} r}_{0}$ increases rapidly.

*The case of* $\omega^{2} LC = 4/3$. When $\omega^{2} LC = 4/3$, we can get $Z_{n} \to \infty$. It is known from Eq. (52) that impedance resonance occurs in the equivalent complex impedance $Z_{n}$. Obviously, $\omega^{2} LC = 4/3$ is a very interesting number.

*The case of* $\omega^{2} LC = 1$. When $\omega^{2} LC = x = 1$, there be $\delta = \eta = - 2$ which can be obtained according to Eq. (50), and $(\delta^{n} - \eta^{n} )/(\delta - \eta )$ can be obtained by taking the limit

53 $$\mathop {\lim }\limits_{\eta \to \delta = - 2} \frac{{\delta^{n} - \eta^{n} }}{\delta - \eta } = n\delta^{n - 1} = n( - 2)^{n - 1} ,$$

therefore, we substitute $\omega^{2} LC = 1$ and Eqs. (53) into (52), yielding

54 $$\frac{{Z_{n} (A_{n} B_{n} )}}{{r_{0} }} = 2 + \frac{2(q - 2)}{{2 + (2 - q)n}}.$$

**Figure 8:**
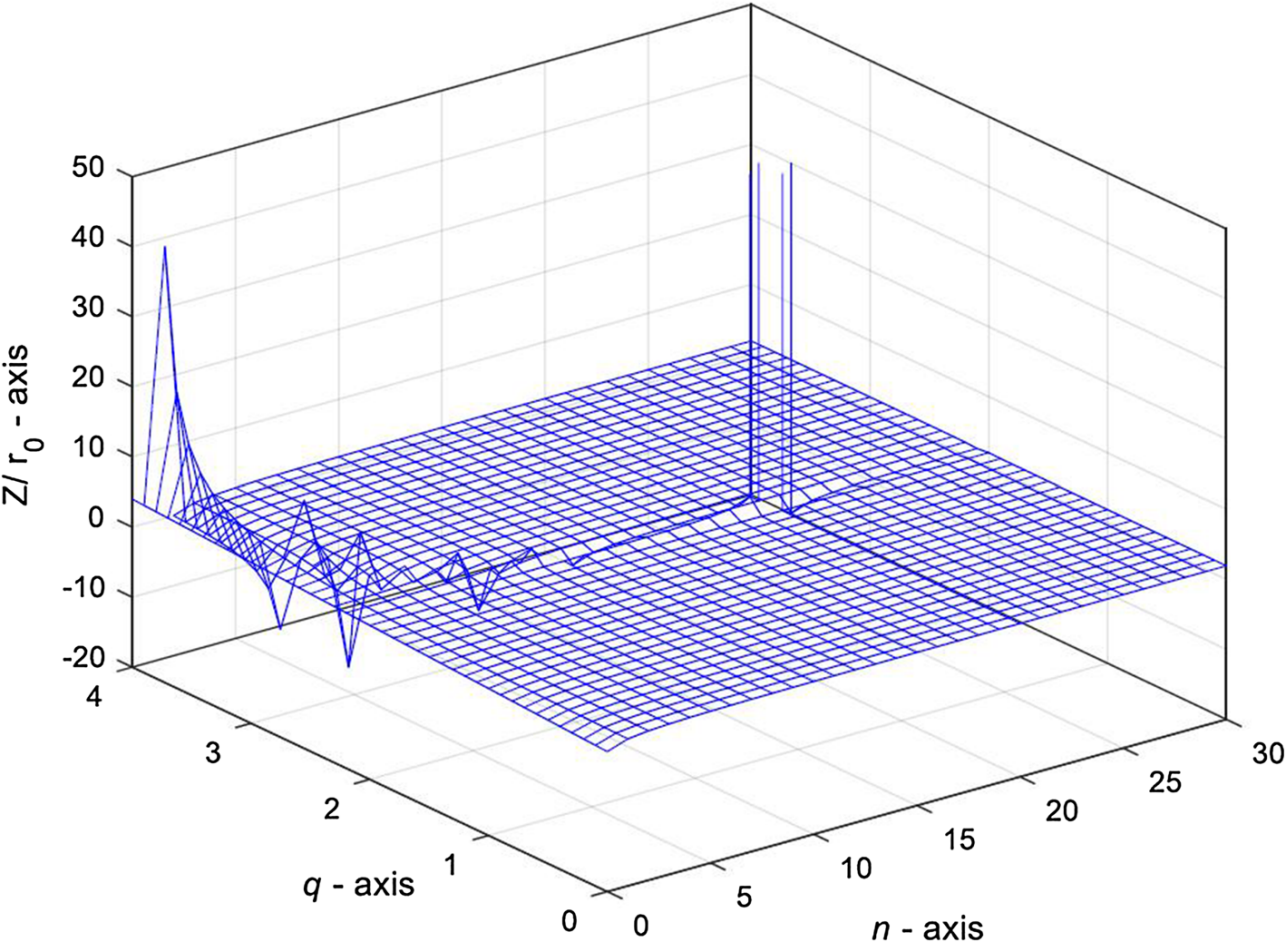
3D graph of the variation of complex impedance ${Z \mathord{\left/ {\vphantom {Z r}} \right. \kern-\nulldelimiterspace} r}_{0}$ with respect to *n* and $q$, in case of $\omega^{2} LC = 1$.

Figure 8 shows that when $\omega^{2} LC{ = }1$, the complex impedance ${Z \mathord{\left/ {\vphantom {Z r}} \right. \kern-\nulldelimiterspace} r}_{0}$ decreases with the increase of $n$ and $q$ as known from Eq. (54) except for the special cases of *q* = 2 and *n* = 0.

*The case of* $0 < \omega^{2} LC < 1$. When $0 < \omega^{2} LC < 1$, its characteristic roots $\delta$ and $\eta$ are imaginary numbers. So complex analysis is needed to carry out on the characteristic roots. We can get from Eq. (50)

55 $$1 - 2x + 2\sqrt {x(x - 1)} = \cos \theta + i\sin \theta ,$$

where $\theta = {\text{arc}} \cos [(1 - 2x)/(8x^{2} - 8x + 1)]$. Substituting (55) into (50), we can obtain

56 $$\begin{gathered} \delta = \frac{2}{4 - 3x}(\cos \theta + i\sin \theta ), \hfill \\ \eta = \frac{2}{4 - 3x}(\cos \theta - i\sin \theta ), \hfill \\ \end{gathered}$$

by substituting (56) into Eq. (52), we can obtain the analytical expression of the equivalent complex impedance of the eigenvalue in the complex case

57 $$\frac{{Z_{n} (A_{n} B_{n} )}}{{r_{1} }} = \phi - \frac{4}{{(4 - 3x)^{2} }} \cdot \frac{{\mu \sin (n\theta ) + \left[ {q - \phi } \right] \cdot \sin ((n - 1)\theta )}}{{\mu^{2} \sin ((n + 1)\theta ) + \mu \left[ {q - \phi } \right] \cdot \sin (n\theta )}},$$

where $\theta = {\text{arc}} \cos (1 - 2x)$, and $\phi = \frac{2(1 - x)}{{4 - 3x}}$ and $\mu = \frac{2}{4 - 3x}$ are defined.

Next, we draw the characteristic curve of equivalent complex impedance at $0 < \omega^{2} LC < 1$ by MATLAB to reveal the variation law of equivalent complex impedance when the eigenvalue is complex in many cases.

**Figure 9:**
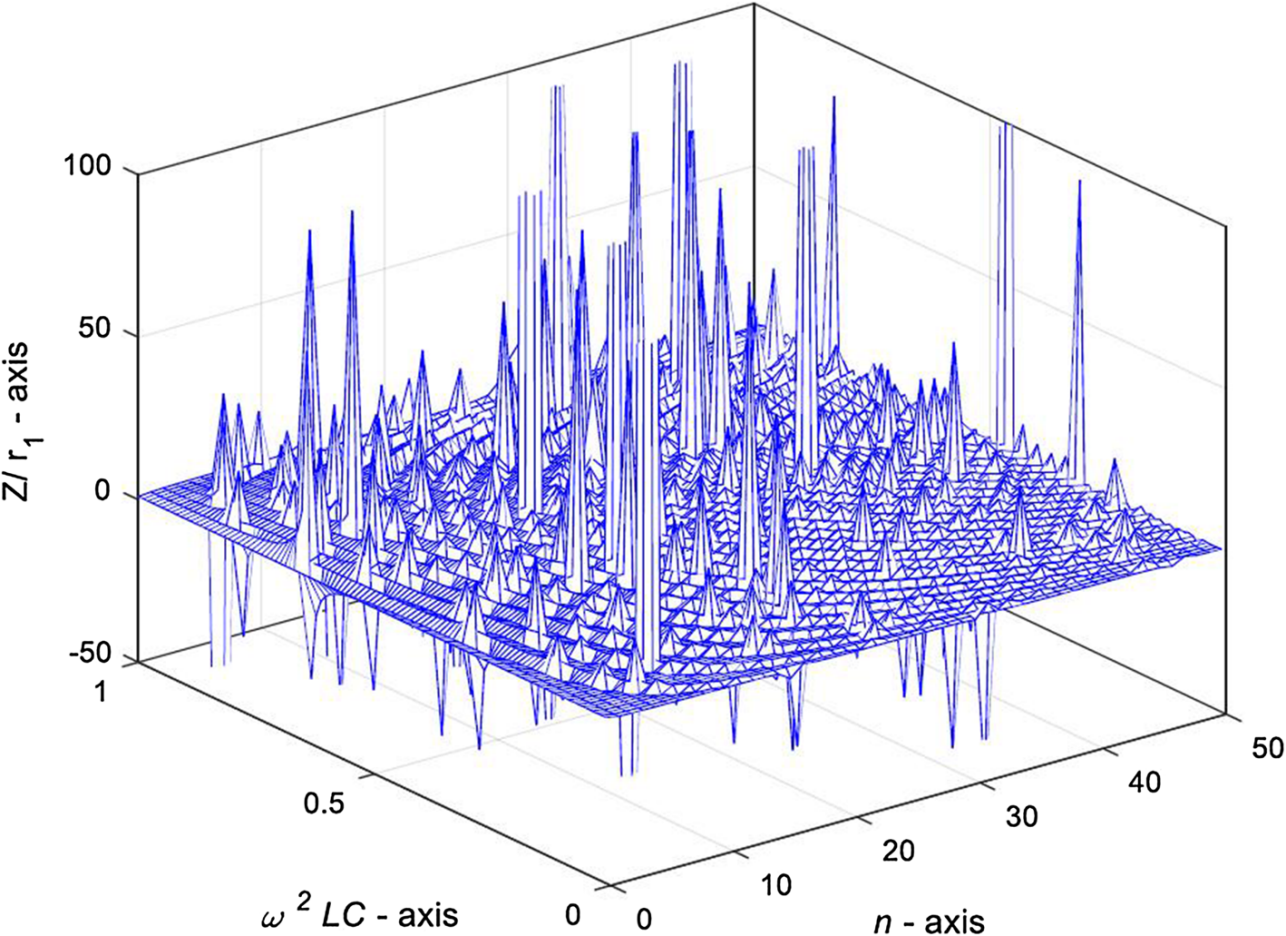
The 3D graph of complex impedance ${Z \mathord{\left/ {\vphantom {Z r}} \right. \kern-\nulldelimiterspace} r}_{0}$ varying with variables $\omega^{2} LC$ and *n*, in case of $0 < \omega^{2} LC < 1$ and $q = 1$.

Figure 9 shows that when $0 < \omega^{2} LC < 1$ and $q = 1$, the complex impedance ${Z \mathord{\left/ {\vphantom {Z r}} \right. \kern-\nulldelimiterspace} r}_{0}$ in Eq. (57) changes irregularly with the increase of $\omega^{2} LC$ and $n$. When $0 < \omega^{2} LC < 1$ and $0 < n < 50$, the complex impedance ${Z \mathord{\left/ {\vphantom {Z r}} \right. \kern-\nulldelimiterspace} r}_{0}$ has irregular oscillation characteristics and resonance characteristics, the oscillation is dense, and the distribution of oscillation amplitude is not obviously regular.

**Figure 10:**
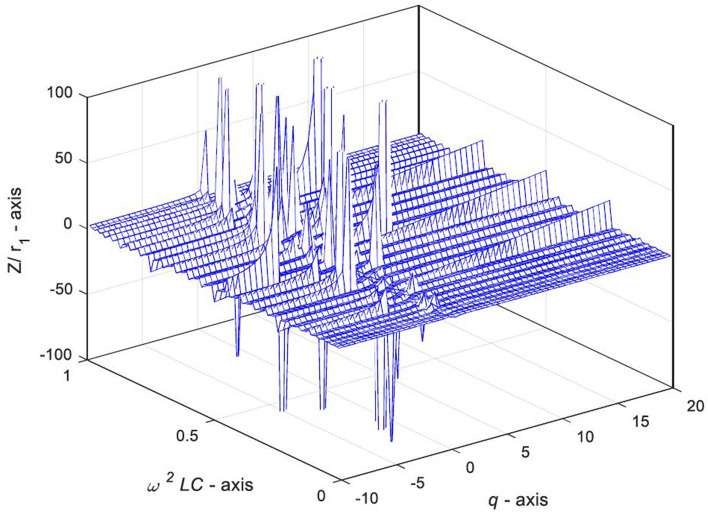
The 3D graph of complex impedance ${Z \mathord{\left/ {\vphantom {Z r}} \right. \kern-\nulldelimiterspace} r}_{1}$ varying with variables $\omega^{2} LC$ and $q$, in case of $0 < \omega^{2} LC < 1$ and $n = 30$.

Figure 10 shows that when $0 < \omega^{2} LC < 1$ and $n = 30$, the complex impedance ${Z \mathord{\left/ {\vphantom {Z r}} \right. \kern-\nulldelimiterspace} r}_{0}$ in Eq. (57) changes irregularly with the increase of $\omega^{2} LC$ and $q$, where, when $\omega^{2} LC$ is a definite value and $q$ is a variable, the complex impedance ${Z \mathord{\left/ {\vphantom {Z r}} \right. \kern-\nulldelimiterspace} r}_{0}$ has regular oscillation characteristics, and the distribution of oscillation amplitude is obviously regular.

### A summary

In this paper, a general *n*-order resistance network model with horizontal bridge circuit (Fig. 1) is proposed, which has not been studied before. The *n*-order recursive transformation method (N-RT method) is used to evaluate the equivalent resistance of *n*-order resistance network with horizontal bridge circuit, and the analytical expression of the equivalent resistance between ${A}_{n}$ and ${B}_{n}$ nodes, Eq. (27), is given by using the solution of the fractional difference equation. Using Eq. (27) can help us continue to analyze and study the equivalent resistance in a variety of special cases. Since the network in Fig. 1 contains five independent parameters, some interesting conclusions are obtained by discussing some cases of equal or limit parameters, for instance, when $r_{3} = r_{1}$, $r_{1} = r$, $r_{3} = 0$ etc. And compared with relevant literature, the results of the present research obtained are correct, which is also due to the self-consistency of our research. As the derivation process and method are rigorous and accurate, the results can be applied to other engineering or scientific aspects in the future. In addition, the research ideas and techniques in this paper can also be employed to study complex impedance, such as the *LC* network shown in Fig. 6. In this paper, the basic characteristics of *LC* network are studied and analyzed in detail, and the characteristic curves of equivalent complex impedance varying with different parameters are drawn. Besides, the theoretical results of this paper have potential application value. For example, the research of Metagratings and super surface material is an important scientific and engineering problem. The research of Metagratings and super surface material may need to be simulated by equivalent circuit model^33,34^.
